# There’s an App for That: A Mobile Procedure Logging Application Using Quick Response Codes

**DOI:** 10.5811/westjem.2020.10.48724

**Published:** 2020-12-10

**Authors:** Jason Folt, Patrick Lam, Joseph Miller, Nikhil Goyal

**Affiliations:** * Henry Ford Health System, Department of Emergency Medicine, Detroit, Michigan; † Henry Ford Health System, Department of Internal Medicine, Detroit, Michigan; ‡ CaroMont Regional Medical Center, Department of Emergency Medicine, Gastonia, North Carolina

## Abstract

Emergency medicine residents are required to accurately log all procedures, yet it is estimated that many procedures are not logged. Traditional procedure logging platforms are often cumbersome and may contribute to procedures not being logged or being logged inaccurately. We designed a mobile procedure logging application (app) that uses quick response (QR) codes to input patient information quickly and accurately. The app integrates with our current procedure log database while maintaining information privacy standards. It scans the QR code displayed for patient identification, automatically extracting pertinent patient information. The user selects the procedure performed and the app uses data analytics to recommend logging other related procedures.

A mobile procedure logging app using QR codes decreases time needed to log procedures and eliminates data entry errors. Improving the speed and convenience of procedure logging may decrease the discrepancy between performed and logged procedures. A similar app can be integrated into any residency program and may improve assessment of resident procedural competency.

## BACKGROUND

Maintaining a complete and accurate procedure log is a fundamental element of emergency medicine (EM) residencies. Such logs assist in the assessment of procedural competency and help identify areas of study during resident self-evaluation.^1^ Additionally, the procedure log is an important Accreditation Council for Graduate Medical Education requirement for EM residencies, yet poor compliance with procedure logging requirements is one of the most frequent citations during accreditation reviews.^2^–^4^ It is estimated that only 37%–60% of performed procedures are eventually logged.^5^, ^6^

Our program’s procedure-logging system (“Website”) has a data entry webpage that interfaces with a secure database. It was developed in-house and resembles popular commercial platforms.^7^, ^8^ It requires accessing a workstation, logging in, selecting a date and procedure, and then manually typing patient name, age, gender, medical record number (MRN), faculty supervisor, and rotation name. This can be cumbersome during a busy work period and may contribute to low logging rates. Some residency programs have developed mobile device-based workflows to mitigate access issues, yet they still require manual data input that may lead to inaccurate logs due to data entry errors.^9^–^13^ Procedure logs are a key component of learner assessment in competency-based medical education; therefore, incomplete logging or erroneous patient information may have substantial implications for the resident, the residency program, and our patients.^1^

## OBJECTIVES

The study goal was to demonstrate feasibility of a mobile procedure logging application (“app”) that uses quick response (QR) codes to automatically read patient data. The primary objective was to compare the speed and accuracy of the app to traditional processes. The secondary objective was to measure app adoption by comparing percentage of app-logged procedures during initial deployment to a similar period three years later.

## DESIGN

A mobile app was created and deployed at a 50-resident EM program via a 10-minute introduction during didactics. Login information for each user is securely stored in the app to eliminate the need for repetitive logins. The app scans the QR code on each patient’s identification sticker and automatically extracts the patient name, birthdate, MRN, and gender. When a user selects the procedure performed from a drop-down menu, the app then suggests other commonly associated procedures that the user may select if they were also performed. For example, the app may say: “Residents who logged I&D also logged ultrasound guidance or procedural sedation; please select the corresponding checkbox to log these.” The procedure or procedures are then recorded in the procedure log database.

The study period was January 1, 2016–March 31, 2016, during which residents had the option to use the app or Website. The three-month period was chosen as consistent with the rapid application development method of software development.^14^ All procedures were logged by residents without additional assistance or direct observation. Every procedure log entry created during the study period was examined. Google Analytics measured the time taken on the app or Website to complete a log entry and then we calculated the mean time taken to log a procedure using each method. We then compared results for the app vs the Website. For the secondary objective, the proportion of total procedures logged via the app during the study period was compared to the proportion logged between January 1, 2019–March 31, 2019.

To identify data entry errors, procedure log patient information was compared to corresponding information in the electronic health record. When the last name, age or gender of the patient in the procedure log did not match the medical record, the data was flagged as an error. MRN errors could not be captured as it was not possible to link these to a unique medical record. Some of these unmatched MRNs may have been errors, but they also may have represented procedures performed at other hospitals while on outside rotations. We excluded these unmatched MRNs from analysis.

Institutional review board approval and informed consent were obtained. The app was developed by one of the authors using HTML, JavaScript, PHP, and MySQL over approximately 10 hours of time. A QR code reader library was purchased for $1600 and the app was deployed on a basic intranet server. Source code for the app is freely available from the authors and may be easily modified to work with any residency’s procedure log database. We used descriptive statistics to compare procedure logging using the app or Website, and chi-square analysis was used for categorical comparisons.

## IMPACT/EFFECTIVENESS

A total of 2930 procedures were logged during the study period, of which 142 (4.8%) were logged using the app by 11 unique residents. On average, it took 27 seconds to log a procedure using the app, compared to 80 seconds using the Website. Data entry errors were significantly decreased using the app compared to the Website (Table 1). All procedures logged by the app were accurate and without errors in patient information.

After three years, there was a fivefold (95% confidence interval 4.3× to 6.0×) increase in the proportion of procedures logged with the app to 841/3397 (24.8%; P<0.001). There were 18 unique resident users during this time. A mobile application using QR codes proved feasible at quickly and accurately logging procedures. The mean time spent logging each procedure substantially decreased, suggesting the app was easier to use. QR codes have previously been used for various applications in healthcare education, but the timesaving and error reduction in procedure logging has not been reported.^15^ The recommendation algorithm for suggesting frequently co-logged procedures is also novel.

The app’s low initial adoption rate increased significantly over time. This may be because of the hospital’s information security requirement to install encryption software on personal phones. With education about privacy implications of this software, app usage increased without any mandate by the residency. Additionally, as senior residents who were comfortable with the Website graduated, incoming residents adopted the app more readily. There do remain other barriers to the app use: Some residents do not carry their phones on shift, limiting their ability to use the app. Additionally, residents rotate at sites outside of our hospital that do not use QR code technology for patient identification.

This study has several limitations. Because it was a feasibility study performed at a single center with a convenience sample, generalizability is therefore limited. Due to privacy concerns, Google Analytics does not allow for analysis of individual procedure logging sessions. We therefore could not calculate variability in the procedure logging time data to detect statistically significant differences. Additionally, the number of unsuccessful logging attempts by the app and the Website was not available. Finally, while we were not able to identify procedures that were performed but not logged, we believe improving the speed, feasibility, and convenience of procedure logging may decrease the discrepancy between performed and logged procedures.

In conclusion, this proof-of-concept shows that a mobile procedure logging application that reads patient information using quick response codes decreases the time to log a procedure and eliminates data entry errors. Compared to traditional procedure logging tools, the app may generate a more accurate record of resident procedural competence. While more rigorous studies are needed to verify these findings, we feel this technology is applicable to other residencies and specialties that require residents to maintain a procedure log.

## Figures and Tables

**Table 1:** Comparison of data entry errors through a program’s online procedure-logging system (Website) compared to a mobile procedure logging application.

Data field	Website errors (rate)	App errors	P-value
Patient last name	374 (15%)	0	<0.001
Patient age	237 (9%)	0	<0.001
Patient gender	60 (2%)	0	0.074

*App*, application.
